# A Lightweight and Low-Power UAV-Borne Ground Penetrating Radar Design for Landmine Detection

**DOI:** 10.3390/s20082234

**Published:** 2020-04-15

**Authors:** Danijel Šipoš, Dušan Gleich

**Affiliations:** Faculty of Electrical Engineering and Computer Science, The University of Maribor, Koroska Cesta 45, 2000 Maribor, Slovenia; dusan.gleich@um.si

**Keywords:** landmine detection, SFCW, ground penetrating radar, low-power, lightweight, UAV, drone

## Abstract

This paper presents the development of a lightweight and low-power Ground Penetrating Radar (GPR) to detect buried landmines in harsh terrain, using an Unmanned Aerial Vehicle (UAV). Despite the fact that GPR airborne systems have been already used for a while, there has yet been no focus on the UAV autonomy, which depends on the payload itself. Therefore, the contribution of this work is the introduction of a lightweight and low-power consumption GPR system, which is based on the Stepped Frequency Continuous Wave (SFCW) radar principle. The Radio Frequency (RF) transceiver represents an improved implementation of the super-heterodyne architecture, which currently offers higher sensitivity. This is achieved by combining analog and digital processing techniques. The experimental results showed that the developed system can detect both metallic and plastic buried targets. Target detection with a scanning height up to about 0.5 m shows good applicability in an unstructured, harsh environment, which is typical of mined terrain. The proposed system still needs some improvements for a fully operational system regarding different aspects of scanning speeds and soil properties such as moisture content.

## 1. Introduction

Humanitarian de-mining is still a current problem due to the amount of buried landmines, where hard accessible areas additionally slow down the de-mining process. The latest report [1] reveals there are still more than 110 million landmines buried over 59 states and other areas, which daily either injure or kill more than 20 people, of which half are children. Although each year more than 130,000 landmines are cleared, there are more than 100 km${}^{2}$ contaminated.

De-mining can be performed in different ways. Still, the most used device for de-mining is a hand-held detector, where an operator scans affected areas manually and, consequently, risks his life. In practice, the investigated areas are difficult to access, which additionally makes the procedure both slower and dangerous. With classifying landmines to groups of metal and plastic, the technology used in conventional detectors is mostly only able to detect metal landmines. This is due to the fact that most of the devices use Metal Detectors (MD), which take advantage of the induced magnetic field of landmine metal content. In plastic landmines, only a small amount of metal content is present (usually only the trigger), therefore, their detection with an MD is nearly impossible. New techniques that use Electromagnetic Induction Spectroscopy (EMIS) may overcome this limitation and provide better sensitivity [2]. However, the detection is limited by distance, since the sensor has to be as close as possible to the ground surface. Another used technology that has existed for many decades and was previously used for fields such as archaeology, construction, road inspections, etc. is Ground Penetrating Radar (GPR) [3]. Results have revealed that great success was achieved in landmine detection with the use of GPR. Moreover, it currently shows most potential among other technologies [4], where the main benefit is the ability to detect metal and plastic landmines down to the smallest size. The most common systems which use GPR are hand-held devices [5], ground vehicles [6] and, recently, also Unmanned Aerial Vehicles (UAVs) [7]; or even a combination of them [8]. The GPR detection principle is straight forward. It is based on transmitting and receiving electromagnetic (EM) waves using antennas. EM waves reflect from metal, or transition between different dielectric materials (as is the case between soil and the landmine explosive, which is considered as the target). If a backscattered signal is received by the receiving antenna and the amplitude of the bounced signal is above the receivers noise floor, this change can be detected.

As stated, UAV GPR systems already exist and they are gaining in popularity. The technology of UAVs has been developing rapidly. With data fusion of different sensors, they form a robust and smart flying platform, which operates autonomously on pre-defined tracks and avoids potential obstacles. By including the latest geo-location technology such as Real-Time Kinematic (RTK) GPS systems, localization is improved to centimeter-level accuracy [9].

Different GPR systems have been introduced on UAVs for landmine detection. The improvement in Software Defined Radios (SDR) has shown that, besides the major use in communications, high bandwidth boards also open the possibility of short-range radar implementation for UAV use [10,11]. The SDRs can provide a quick confirmation for the proof of concept, but, in many cases, it has a disadvantage in efficiency and mobility. To overcome this, several works have applied commercial equipment or custom-developed GPR systems for efficient buried object detection with GPR and UAV combination [12,13,14]. However, since UAVs are battery-powered devices, the main contributions of this paper are: (i) Decreasing the weight and power consumption of the complete radar system, with the purpose to extend the flight time, or allow use of a smaller UAV platform; (ii) Improved Radio Frequency (RF) front-end sensitivity of the proposed transceiver, which enables a decrease of the RF transmitting power and power consumption. A Stepped Frequency Continuous Wave (SFCW) radar was designed using an improved super-heterodyne architecture of a transceiver [15] by combining analog and digital design techniques. This allowed us to develop a complete radar system on a small PCB board, with independent operation. The radar, with a combination of a suitable antenna setup, forms a complete GPR system, making landmine detection possible even in a harsh environment where the surface is covered with vegetation.

Apart from the radar’s sensitivity, the operating frequency has the highest impact on the coupling of the signal with the propagation medium. Previous contributions [16,17,18] have shown that, for short-range GPR, lower frequency bands (such as the S, L, and UHF bands) are most suitable. The operating frequency also defines the Bandwidth (B) which is related to the range resolution $\Delta R=v_{m}/2B$, where $v_{m}$ is EM velocity in a given medium. This leads us to conclude that, for a GPR, these are some of the main parameters.

Furthermore, the success in landmine detection relies not only on the GPR system itself but as well on the measuring technique. As described in [19,20,21,22] the Forward-Looking GPR (FL-GPR) method was used on vehicles as well as on UAVs, where there is a certain angle between the ground and the GPR antennas. Other recent work uses the Down-Looking GPR (DL-GPR) method, where antennas are positioned perpendicular to the investigated surface [23]. For the proposed system, the DLGPR method was selected, due to the fact that it requires less transmitting power since the antenna beam is perpendicular to the ground. Maximal reflection is achieved by selecting this method. Considering the mentioned parameters, the developed system can extend the battery autonomy with no loss in performance, or be used on smaller sized UAV systems and, consequently, lower the total system cost.

## 2. Stepped Frequency Continuous Wave Radar

### 2.1. Principle of Working

SFCW Radar has been known for many decades [15]. The transmitter generates a synthetic pulse with *N* frequencies, spaced with a uniform step of Δf over time $t_{PRI}$, which is the Pulse Repetition Interval (PRI). The frequency of each step is given by

(1) $$f_{n}=f_{0}+n\Delta f\;n=0,1,2,\ldots ,N,$$

where $f_{0}$ is the starting frequency. The bandwidth of the radar system can be defined as B=NΔf and is correlated directly with the range resolution given by

(2) $$\Delta R=\frac{v_{m}}{2B},$$

where $v_{m}$ is the EM propagation speed in the propagation medium. Since ΔR is constant *N* defines the maximal range given by

(3) $$R_{max}=\Delta RN.$$

A target at a certain distance causes that on the RF front-end a two-way time-delayed version of the transmitted signal over time *t* is received, and is given by

(4) $$s_{n}\left(\omega_{n},t\right)=A_{n}\exp\left(-j\left(\omega_{n}\left(t+\tau \right)+\theta_{n}\right)\right),$$

where $A_{n}$ represents the amplitude of the reflected signal, $\omega_{n}=2\pi f_{n}$, τ represents the two-way time delay in propagation space, and $\theta_{n}$ the phase offset. To avoid high-speed ADCs, the signal can be down-modulated to a lower Intermediate Frequency (IF), and later demodulated to an In-phase and Quadrature (IQ) base-band signal, which keeps the magnitude and phase ratio information of transmitted and received signals. The IQ base-band signal for all frequencies is given by

(5) $$C_{n}\left(\omega_{n},\tau \right)=I_{n}\left(\omega_{n},\tau \right)+jQ_{n}\left(\omega_{n},\tau \right)=\exp\left(-j\omega_{n}\tau \right)=\exp\left(-j\phi_{n}\right),$$

where a complex vector *V* corresponds to one range bin of size *N*, given by

(6) $$V=[C_{0},C_{1},\ldots ,C_{N-1}].$$

A pulse response $y_{n}$ within the time domain is obtained by using an Inverse Discrete Fourier Transform (IDFT) of received elements of complex valued vector *V*

(7) $$y_{k}=\frac{1}{M}\sum_{i=0}^{M-1}C_{i}\exp\left[\frac{j2\pi ki}{M}\right],$$

where 0≤k<M. To increase the resolution, complex vector *V* has to be extended with zero padding to size *T* before applying the IDFT and taking the magnitude gives

(8) $$|y_{k}|=\left|\frac{1}{T}\sum_{i=0}^{L-1}C_{i}\exp\left[\frac{j2\pi ki}{T}\right]\right|.$$

If a target were present the result would be visible as a synthetic pulse on the location corresponding to the caused time delay.

### 2.2. Sfcw Radar Advantages

The radar systems differ in operation principles and system performances. In this paper, an application of the SFCW radar principle with a super-heterodyne architecture is presented, and has the following main advantages compared to other frequency domain radars: (i) High-frequency bandwidth at lower frequency bands compared to the well-known Frequency Modulated Continuous Wave (FMCW) radar [24]. The FMCW radar method requires the generation of a continuous linear sweep, which is mostly generated with a Voltage Controlled Oscillator (VCO). The sweep is continuous, therefore, no interruption can be caused to switch between multiple VCOs, and the bandwidth is limited by the single VCO frequency range. The SFCW method has the advantage that the frequency sweeping is generated in steps, which means that multiple VCOs can be used with a combination of frequency dividers to set the desired frequency for each step. This procedure is explained in detail in Section 3.2. (ii) Higher harmonics components or other amplitude variations of the transmitted signal do not affect the base-band signal at the receivers site. This is due to the selected super-heterodyne architecture, where the IF signal always has a fixed frequency, meaning that a highly optimized filter can be designed for the specific IF frequency. Instead, in the FMCW radar, the base-band frequency changes linearly with the target distance. Therefore, a low-pass filter has to be used with a certain pass-band. Since closer targets have lower frequencies, this means that their higher harmonics could be still inside the pass-band of the filter and result in aliasing. An option would be to filter the transmit signal in the RF domain, but, because of the wide bandwidth, a single filter does not offer sufficient performance.

Compared to the time domain radars, such as, for example, an Impulse radar, an advantage is avoiding hardly available components. Namely, pulse generators, in most cases, include components such as Step Recovery Diodes (SRD) [25] which can, nowadays, barely be found. Furthermore, slight parameter drifts from one to another of these components can influence the overall radar performance greatly. The receiver of an Impulse radar requires a high-speed ADC (a range of more than >1 Gsps) which is expensive; or a sub-sampler, where the signal must remain the same for the time that the repetitive acquisition is undergoing. Additionally, for Impulse radars, only a limited number of antennas is suitable, since many of them have dispersive characteristics.

The SFCW radar with super-heterodyne architecture has great overall performance, however, it also has a disadvantage compared to all other methods, which is the acquisition speed. This is affected due to the switching between frequencies, and the detailed effect will be explained in Section 4.2.

## 3. Sfcw Radar Design

**Figure 1:**
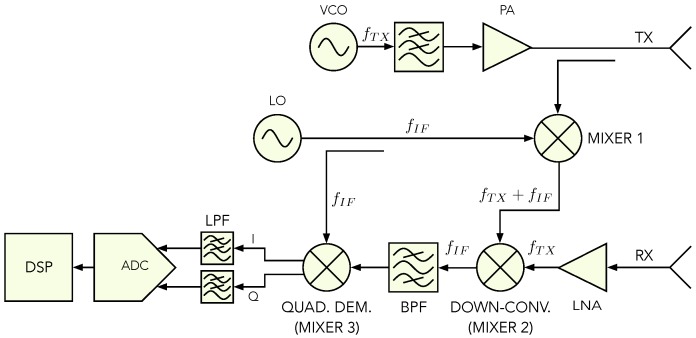
Block scheme of super-heterodyne Stepped Frequency Continuous Wave (SFCW) radar [15].

**Figure 2:**
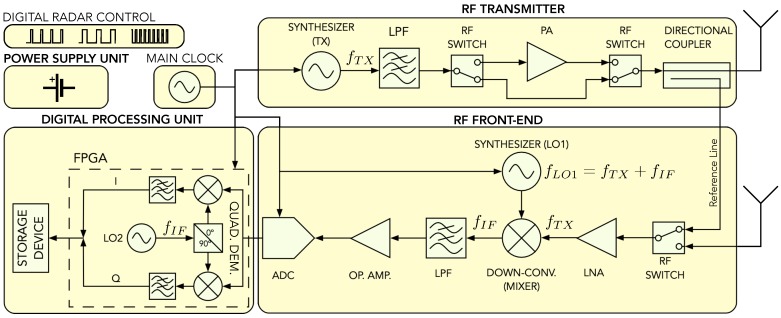
Block scheme of the proposed SFCW Ground Penetrating Radar (GPR) board with indicated main system parts.

The SFCW radar principle can be based on a homodyne or a super-heterodyne architecture. Both architectures follow the same principle, which was explained in the previous section, but the super-heterodyne architecture shows better performance, since the RF input signal is, firstly, down-modulated to an IF signal. This signal can be then filtered from higher harmonics easily, since they could cause ghost targets. Figure 1 shows a basic block scheme of the super-heterodyne architecture. However, the structure still contains crucial disadvantages which have an impact on the Signal-to-Noise Ratio (SNR): (i) The design consists of three mixers which, compared to other RF components, add most of the noise to the system; (ii) a dual-channel ADC is required to sample the I and Q channels; (iii) the quadrature detector (mixer 3) additionally causes phase noise. The proposed design of the radar uses a single mixer, an ADC with a single channel input, and transferring the quadrature demodulation from the analog to the digital domain, as shown in Figure 2.

In general, the system is divided into a power supply, transmitter, RF front-end and digital processing unit. A main clock source is used to ensure synchronization between all units. Under the supply unit, different voltage levels are provided to other blocks. The main purpose of the transmitter is to generate the transmitting signal and reference signal which is obtained by a directional coupler. The entire transmitter structure will be described further in Section 3.2. The transmitter and RF front-end block use identical frequency synthesizers (TX and LO1), with a constant frequency difference $f_{IF}$ to generate an IF signal. The RF front-end will be described in detail in Section 3.3. After the ADC, all further digital processing is realized on a Field Programmable Gate Array (FPGA). Data can be transferred to a PC over a USB connection, or stored internally on a memory device. In the same way, radar parameters can also be uploaded to the radar from either a PC or a memory device. The radar also includes digital input pins for external commands.

### 3.1. Air-Launched Gpr Antenna

Selecting the correct antenna for GPR systems depends on the type of measurement. The antenna can be either coupled directly with the ground surface (ground-coupled system) or be located at a certain distance from the ground surface (air-launched system). Since in ground-coupled systems only one propagation medium is present, less care is needed in the antenna selection. A wide range of antennas is suitable for this task, especially planar antennas. The proposed method, which includes a UAV, requires the use of an air-launched system. In this scenario, the signal propagates through two main mediums (air and soil), where, after each transition, reflection occurs. This consequently then also lowers the target amplitude. Furthermore, achieving reasonable coupling across a wide frequency band with a starting frequency at sub-GHz is a challenging task, especially if the coupling medium is air. This is due to the high impedance mismatch of radar feed line (in most of cases 50 Ω) and air characteristic impedance (approximately 377 Ω). The antenna has to resolve this transition gradually to avoid reflections already inside the antenna.

**Figure 3:**
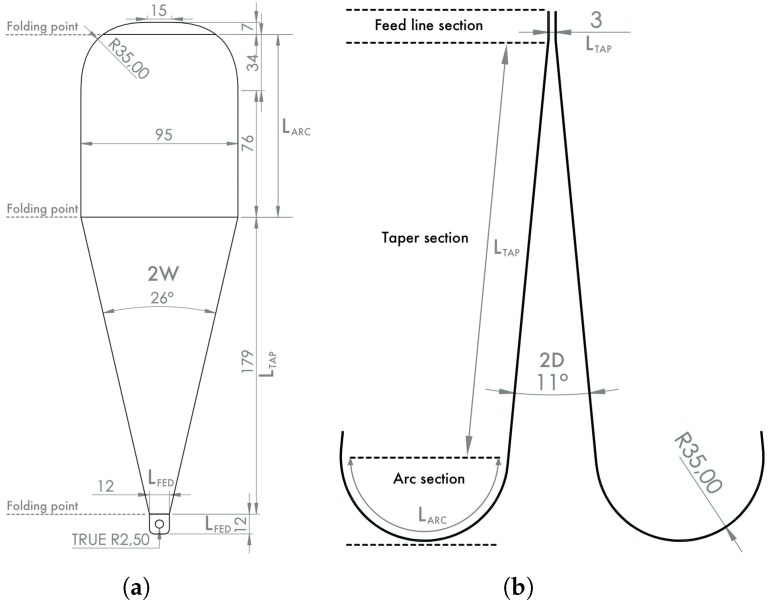
(**a**) Top view of the VH antenna dimension, when the part is flattened; (**b**) side view of antenna assembly and given flattening parameters (the given values are expressed in mm) [26].

**Figure 4:**
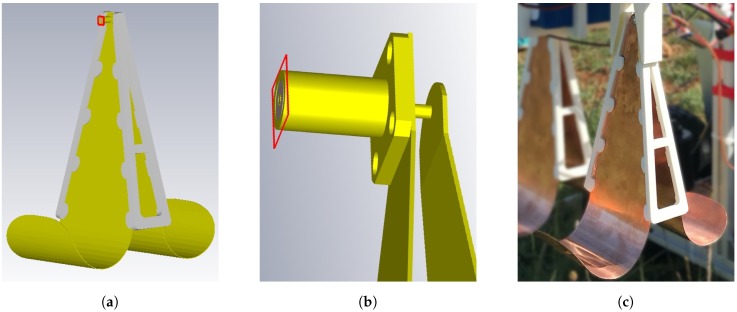
(**a**) Vivaldi–Horn (VH) antenna and a form holder (PLA polymer material with permittivity *ε_PLA_* = 1.8 at 1.5 GHz [27]) simulation model, which design is based on dimensions shown in Figure 3; (**b**) close up view of the antenna feed point (solid red line); (**c**) developed VH antenna with a 3D printed form holder.

**Figure 5:**
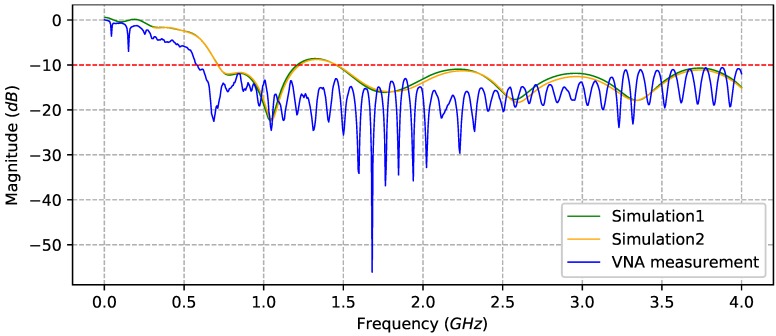
$S_{11}$ scatering parameters of simulated VH antenna without form holder (solid orange line), with form holder (solid green line), and measured VH antenna (solid blue line). The dashed red line indicates the limiting magnitude from where the antenna is suitable for the selected frequency.

**Figure 6:**
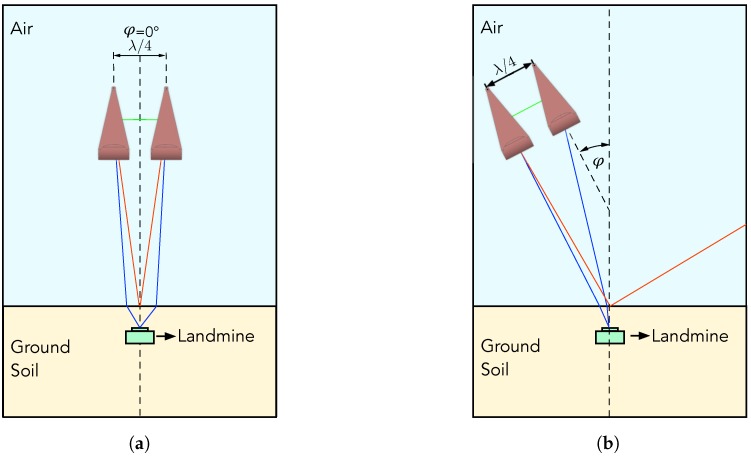
(**a**) Down-looking GPR (DL-GPR); (**b**) forward-looking GPR (FL-GPR).

Vivaldi antennas are used widely for systems with limited weight and size, because they can be designed easily using a thin copper film on a planar substrate. The GPR systems use lower frequency bands, which, consequently, increase the antenna size. A hybrid Vivaldi–Horn (VH) antenna design was proposed in [26], and used for impulse-based radars. It shares the benefits of both Vivaldi and Horn antennas, and it transforms the planar Vivaldi antenna to a 3D space, where the major benefit could be found in the smaller antenna size. The antenna input impedance $Z_{in}$ is related with the output impedance $Z_{out}$ as

(9) $$Z_{in}=Z_{0}\frac{Z_{out}+jZ_{0}tan\left(\beta l\right)}{Z_{0}+jZ_{out}tan\left(\beta l\right)},$$

where $Z_{0}$ is the characteristic impedance, *l* is the feed line length and β is the wavenumber of the transmission line. The Equation (9) is a simplified model. To obtain optimal performance, the transition must be divided into the following sections: Feedline section, taper section and arc section with dimensions shown in Figure 3. This antenna design was originally proposed in [26]. The antenna performance was, firstly, simulated in the CST Studio Suite software using the design shown in Figure 4a, where apart of the waveguide (yellow) an additional PLA polymer part (white) is used to maintain the form of the waveguides. To evaluate the operating frequency band, the magnitude of S${}_{11}$ scattering parameters should remain below −10 dB. Simulation results are shown in Figure 5, where the solid orange line shows the result without form holder and solid green line with form holder. The frequency limit has been set to 4 GHz, since higher frequencies are not efficient for GPR use. Results reveal that the influence of the antenna form holder is minimal.The antenna was fabricated out of a thin copper plate with thickness d=0.5 mm and feed over an SMA connector, soldered to the antenna (Figure 4c). The antenna outbound size was 95 mm × 225 mm × 180 mm with a weight of 240 g, which would also make it suitable for middle payload UAVs. To validate the simulations, the developed antenna was measured with the use of a Vector Network Analyzer (VNA). All results are compared in Figure 5. The measurements and simulations (with form holder) mismatch slightly in the lower frequency band, but otherwise remain below the limiting magnitude at the same range. Furthermore, since the air-launched method of scanning was chosen, the angle between antenna and ground surface also influences the backscattered signal. The angle can be either (i) $\varphi =0^{\circ }$, so that it is considered as a down-looking GPR (DL-GPR) system (Figure 6a); or (ii) $\varphi >0^{\circ }$, where the system becomes front-looking GPR (FL-GPR) (Figure 6b). In practice, the impact in the design of GPR RF will depend on the selected method. In the previous section, the scenario of one target has been described according to Equation (4). Considering a real environment, the signal results in the sum of all reflected targets as:

(10) $$s_{n}\left(\omega_{n},t\right)=\sum_{m=1}^{K}A_{n,m}\exp\left(-j\left(\omega_{n}\left(t+\tau_{m}\right)+\theta_{n}\right)\right),$$

where *K* is the number of targets and $\tau_{m}$ is the time delay of the corresponding target. In Figure 6 are also shown the typical signals obtained by the receive antenna. By ranging them by their magnitude value from strongest to weakest, they have the following order: Direct antenna coupling (solid green line), ground reflection (solid orange line) and landmine back-scatter (solid blue line). The sequence depends on the number of propagation mediums that the signal has to travel from one to another antenna; observation angle; the relative antenna-to-antenna location. By selecting an FL-GPR the ground reflection decreases greatly, although higher transmitter power is needed because the target is observed under a certain angle. Since, for the proposed system, the priority was low power consumption, the DL-GPR method was selected.

### 3.2. Transmitter Design

The main purpose of the transmitter is to provide a signal with equal and sufficient power through the whole used frequency band. Continuous Wave Radars generate frequency sweeps either linearly (FMCW radar) or in uniform steps (SFCW radar), normally using a VCO. Considering the need to cover the full bandwidth, the latest technology does not allow the generation of such a wideband frequency sweep within the selected frequency band. Accordingly higher bandwidths can be obtained with use of multiple VCOs at higher frequencies. Afterwards, with a combination of frequency dividers, the output frequency of selected VCO can be scaled to the desired band. A frequency synthesizer was used in an IC chip, which also included a Phase Locked Loop (PLL) to match the corresponding frequency exactly. The frequency synthesizer allowed us to generate a frequency sweep from 23.5 MHz up to 6 GHz, where the output was limited by the RF front-end components, which are described in Section 3.3. After the synthesizer, a low-pass filter was used in series, followed by an optionally selectable PA. Since each frequency setting requires some time to configure and lock, this does not allow the generation of a continuous linear sweep, and, therefore, an SFCW method had to be chosen. Before the signal is coupled to the transmitting antenna, a directional coupler is used in order to provide a reference signal. The reference signal is directly connected to the receiver and it serves as a reference since the waveguide has a known length. By default, the output power is −4 dBm with disabled PA, or 15 dBm otherwise.

### 3.3. Digital Processing Block and Rf Front-End Design

**Figure 7:**
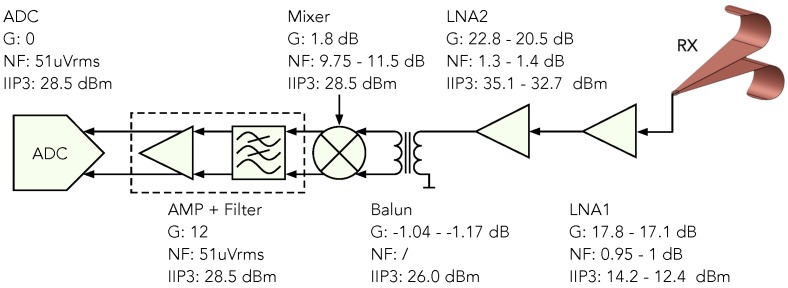
SFCW GPR detailed Radio Frequency (RF) front-end block scheme.

According to the chosen air-lunched DL-GPR method, the RF front-end design also depends on this. Target backscatter signals have significantly lower amplitudes compared to the ground reflected signals, therefore, the performance is highly dependent on the receiver dynamic range. Additionally, the ground reflected signal (which is normally the strongest) has to be just below the receiver saturation level, which ensures coverage of the entire receiver range. Furthermore, the RF front-end must have the ability to filter higher harmonics of the transmitted signal which are causing ghost targets, and they are a very well known problem in this type of radars. To meet all requirements, the following line-up was made, as is shown in Figure 7.

If a target is present, the backscattered signal is amplified using two Low Noise Amplifiers (LNAs) in series. They ensure sufficiently amplifying the received signal, and, at the same time, they have high maximum input power. To prevent cases where too high RF input power could damage further components, LNA2 has the ability to bypass the signal.

The presented design is based on a super-heterodyne structure, used for the purpose of correcting the transmitter imperfections such as the high amplitude of higher harmonics (TX synthesizer 2nd harmonic −40 dBc and the 3rd harmonic −34 dBc). Instead of using only one demodulator (Homodyne structure) to obtain the IQ base-band signal, this structure contains an additional demodulator. The first demodulator converts the high-frequency signal into a lower IF signal, whose frequency was chosen to be $f_{IF}=2$ MHz. The frequency on the LO input of the demodulator is $f_{LO1}=f_{TX}+f_{IF}$, and it is generated with a frequency synthesizer. A bandpass filter is used to remove the higher harmonics of the IF signal $s_{IF}$ which would cause ghost targets. The filtered signal is amplified with an operational amplifier and then sampled directly using an ADC at $f_{s}=40$ Msps. Further processing is carried out digitally using the FPGA. A continuous cosine and sine signal with frequencies $f_{IF}$ are generated out of a lookup table and serve as a source for the digital quadrature demodulator to obtain the IQ base-band signal. In other words, each of the generated signals is mixed with the acquired input signal. This method reduces the number of components and improves the performance, since only quantization noise is added. To improve SNR additionally, each component (I and Q) is sampled for $N_{IQ}$ iteration, and passed through a mean filter. Since the main clock source is shared between all the blocks, there is no phase drift. The generated signal at a specific frequency step has an unknown phase due to the chosen frequency synthesizer properties. In order to compensate a randomly generated phase shift between LO1 and TX the following steps are performed in the following order:

1. The RF front-end input is coupled with the RX antenna.
2. The IQ base-band signal is acquired, serving as the receiver IQ base-band data $O_{n}$.
3. The RF front-end input is directly coupled with the TX channel.
4. The IQ base-band signal is acquired, serving as the reference IQ base-band data $P_{n}$.

As soon as $P_{n}$ is acquired, the absolute IQ base-band signal can be calculated as $C_{n}=\frac{P_{n}}{O_{n}}$, and represents the actual magnitude ratio and phase difference, between transmitted and received signals, caused by a target at frequency *n*.

## 4. Hardware Overview

### 4.1. Uav Overview

To carry the SFCW GPR system, a commercial UAV was chosen [28], which has a flight time up to 18 min with a maximum payload of 5.5 kg. Since the SFCW GPR system weight is far below maximal weight, later, a smaller UAV could also be selected. The control of both geological positioning and guidance is managed by the flight controller itself, and it does not need any other modifications. Furthermore, with the use of the open-source Software Development Kit (SDK) provided by the manufacturer, the SFCW GPR is connected to the flight controller, and it accepts digital inputs to trigger scanning remotely. Telemetry data is available, such as GPS location, velocity, attitude, etc. and it is stored on-board during scanning.

For possible failure scenarios six rotors ensure a safe landing, even if up to three of them are disabled. The same redundancy is achieved using six batteries connected in parallel for a possible failure scenario.

### 4.2. Sfcw Gpr Overview

**Figure 8:**
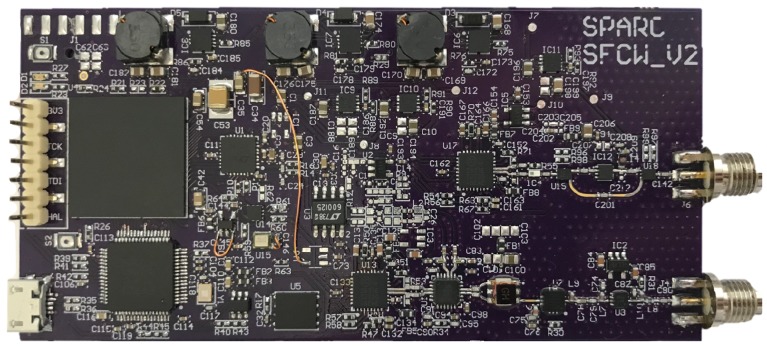
Developed SFCW GPR board.

The main SFCW GPR system was assembled on a single 4-Layer substrate of size 100 mm × 500 mm and it is depicted on Figure 8. Due to the possibility of additionally lowering the total cost, the directional coupler and RF switch were assembled on a separate board of size 20 mm × 30 mm. A 3 cell Lithium-Polymer battery with 1300 mAh of capacity was used for supplying the system. The consumption of the SFCW GPR was measured to be 350 mA, which corresponds to 4.2 W and provides an operation time of more than 3 h.

Not only does the hardware specification influence the radargram quality, but also the GPR parameter settings. One of the most important parameters is the number of IQ base-band signal samples $N_{IQ}$ and the number of frequency steps *N*. These parameters define the time $t_{bin}$ which is needed to capture one range bin and it is given by

(11) $$t_{bin}=\left(2N_{IQ}\frac{1}{f_{s}}+t_{sdel}\right)N,$$

where $t_{sdel}=180$μ is and represents the system delay caused by the time needed for configuring chips and storing data. A value of $N_{IQ}$ lowers the noise, since more samples are averaged, but obviously extends the total time. The number of frequency steps has also the same influence. Increasing this value will increase the Signal-to-Noise Ratio (SNR) of the radargram and extend the maximal range. Therefore, the fact of selecting the parameters and finding an optimal relation between time, resolution and noise is highly important.

The GPR system starting frequency was selected to be $f_{start}$ = 550 MHz, according to the limitations of antenna and LNAs. The upper limit was set only by the LNA1 bandwidth, and it was set to be $f_{stop}$ = 2.7 GHz. Finally, the maximum available bandwidth was B = 2.15 GHz. Since the SFCW GPR frequency band overlaps with the frequency band of the UAV, the PCB board was enclosed into a thin 3D printed plastic case, which was covered with thin metal shielding and ensured isolation. The final SPCW GPR and UAV parameters are summarized in Table 1.

## 5. Experimental Results

The performance and efficiency of the developed radar system are presented in this Section. In order to prove the concept of the developed SFCW GPR system, measurements were first performed in a laboratory environment, and later on an outdoor polygon. For all measurements, the proposed SFCW GPR system was used, where, additionally, different radar parameters were under investigation. A motorized rail was used in the laboratory for simulating a UAV flight path. After proof of concept, the system was attached to a commercial UAV, where measurements were obtained on Anti-Tank (AT) and Anti-Personnel (AP) test landmines. To improve acquisition speed, all unnecessary processing was performed later on the PC. In GPR radargrams background subtraction was performed to remove clutter and improve the image quality.

### 5.1. Laboratory Experimental Tests

**Figure 9:**
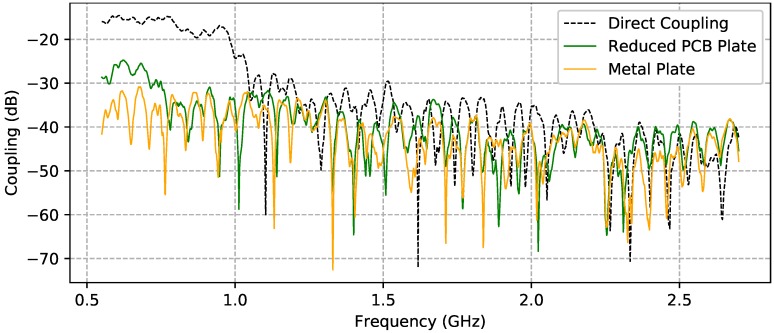
Direct antenna coupling under different conditions. Direct coupling (black dashed line), metal plate (solid orange line), and PCB plate (solid green line).

**Figure 10:**
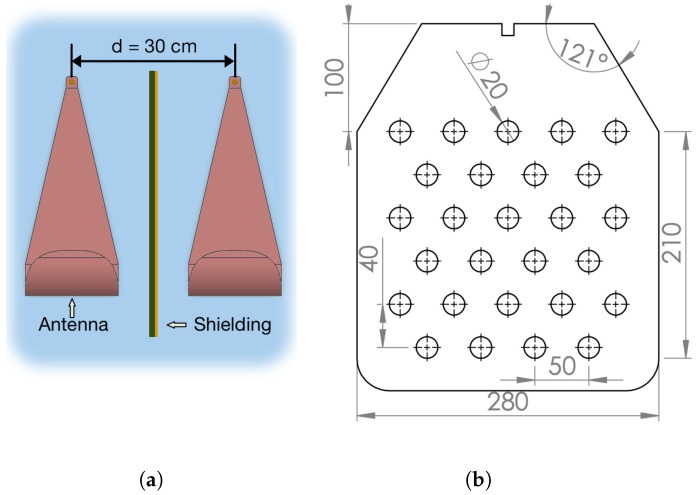
(**a**) Final antenna arrangement with separation of d = 30 cm; (**b**) detailed design parameters of the PCB shield (given values are expressed in mm).

Preliminary measurements had to be performed before using the GPR system on the outside polygon. For this reason, the antenna distribution has been under investigation to find a suitable antenna to ground distance and also GPR parameters. Antenna distribution has a great impact on the final radargram, since the RF front-end is highly sensible and it could saturate the ADC or some other components in the series. The antennas were mounted parallel to each other and separated by a distance d = 30 cm, where the minimum antenna distance rule $d_{ant}>\lambda_{c}/4$ was satisfied. Moreover, according to Equation (10), the direct antenna coupling was also under investigation. As is shown in Figure 9 (black dashed line) direct coupling ($S_{21}$ scattering parameters) is in the range between 550 MHz to 1 GHz by more than 20 dB higher compared to other values. This amount of power would saturate the receiver in this band and, consequently, lower the detection capabilities, since lower frequencies penetrate better. The coupling could be reduced successfully with use of a metal planar shielding plate, as shown in Figure 9 (solid orange line). The solution was, furthermore, improved on a PCB plate made of FR4 substrate with thickness $d_{s}=1.6$ mm and permittivity $\varepsilon_{s}=4.34$ at 1 GHz. On the shielding, holes have been added to maximize the reduction of weight. Results are shown in Figure 9 (solid green line). Figure 10 shows the final antenna arrangement and detailed shield design. This shielding does not perform as well as the metal plate, but it still kept the coupling level sufficiently low.

**Figure 11:**
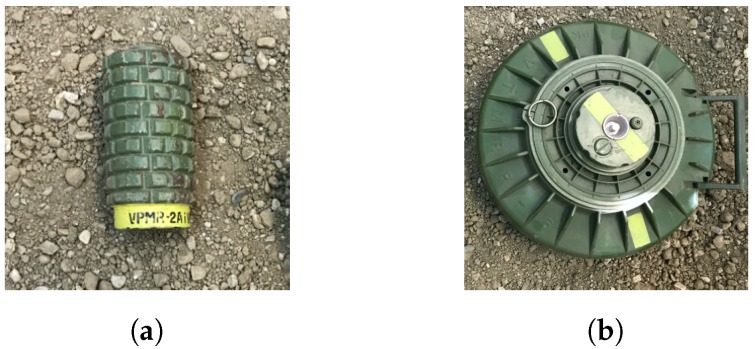
(**a**) Anti-Personnel (AP) metal landmine of size 8 × 17 cm; (**b**) Anti-Tank (AT) plastic landmine of size 27 × 13 cm.

**Figure 12:**
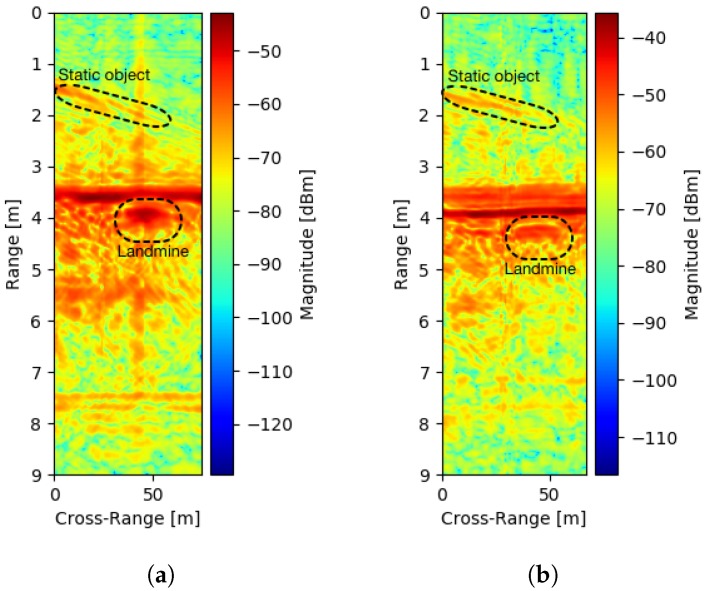
B-Scan with 20 cm deep buried metal AP landmine of size 8 cm × 17 cm in dry mixed soil and antenna to ground distance is 20 cm (**a**) and 45 cm (**b**).

Concerning an air-launched system, the relation between reflected power and antenna distance is also important. The relation may define the antenna to ground distance when the landmine is still detectable. For this measurement, an AP metal landmine of size 8 cm × 17 cm (see Figure 11a) was buried in the polygon box at a depth of 20 cm. A B-scan was then captured using the proposed SFCW GPR mounted to a motorized rail with constant velocity. The whole available bandwidth of B=2.15 GHz was used with *N* = 194 frequency steps. Figure 12a shows a radargram of the lower limit ground distance, selected to be 20 cm, which was enough to avoid smaller ground obstacles. The ground reflected power was −40 dB, and reflected power from the landmine of maximum value −50 dB. In the next scenario, the distance was increased experimentally to the upper ground distance limit of 45 cm with identical parameters, and results are shown in Figure 12b. The ground reflected power remained approximately in the same range, for the reason that the observed ground area had also increased; nevertheless, the landmine reflection decreased by about 15 dB. As seen, the reflection decreased highly with distance, compared to the ground reflection. Therefore, increasing the height of measurement would require a higher dynamic range. In practice, the UAV could fly down to 20 cm in highly flat and clean areas to maximize the reflection of landmines, and up to 45 cm in areas where obstacles are present (e.g., high grass, wasted wood, rocks).From comparing both results, we can also see that when the antenna was closer to the ground, the magnitude increased to a maximum at the landmine center, while, at the other scenario, the magnitude remained almost the same. When the antenna was located further from the ground surface also strong scattering was present from about 3.5 m to 4 m, which is likely because of the border of the box in which the soil is located. On both radargrams, a diagonal line is present, which is caused by a static object located close to the measurement area. Since the developed system was suitable for air-launched measurements, the next Section includes field measurements.

### 5.2. Field Measurements with Use of An Uav

**Figure 13:**
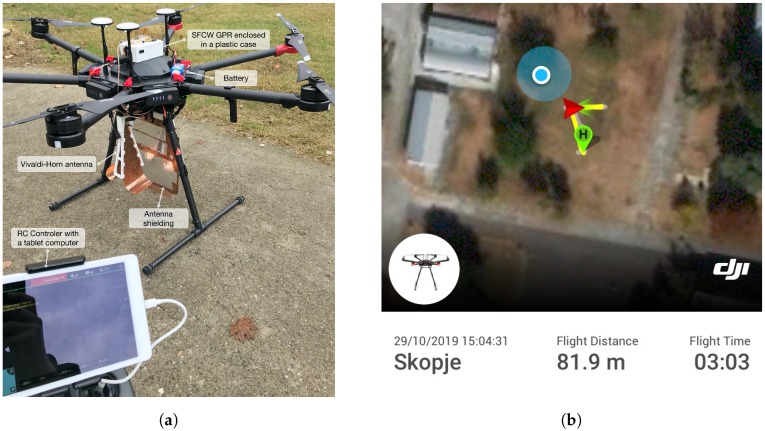
(**a**) UAV and SFCW GPR setup; (**b**) UAV flight path (solid yellow line).

**Figure 14:**
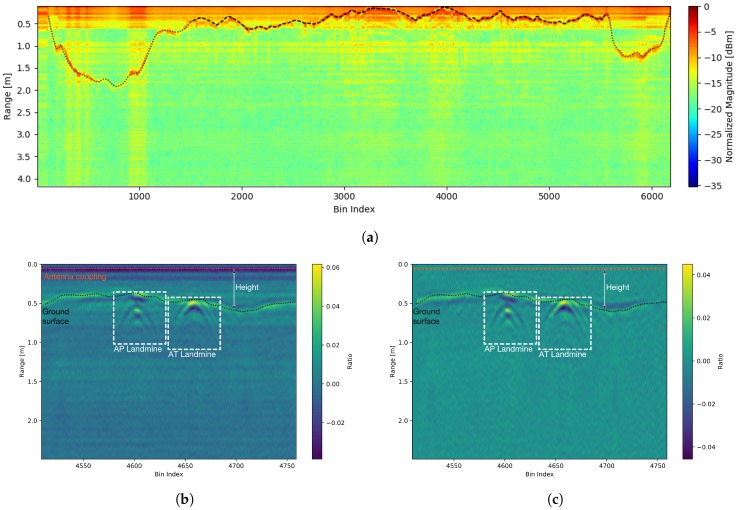
(**a**) Radargram of complete flight with UAV where scanning is visible (dashed black line) and also take-off and landing (dotted black line); (**b**) radargram of basic processed data with depicted landmines (**c**) radargram with background substraction.

The performance of the developed SFCW GPR on a UAV has been validated in a test field in Skopje, North Macedonia, where the final set-up is shown in Figure 13a. Two targets were selected, a metal AP landmine of cylindrical shape, with size 8 cm × 14 cm (see Figure 11a), buried 20 cm deep into the soil, and a plastic AT landmine of cylindrical shape, with size 27 cm × 13 cm (see Figure 11b) with its top aligned to the ground surface. A bandwidth of *B* = 2.15 GHz was used with *N* = 94 frequency steps per range bin. The mapped flightpath is shown in Figure 13b, marked with a yellow line, and the current UAV location. All telemetry data is then available for offline processing, and simplifies the tracking of landmines. The magnitude radargram corresponding to the flight path is shown in Figure 14a. After taking off and allocation to the desired investigation area (dotted black line), the UAV started the scanning procedure with a velocity of about 0.6 m/s at a height between 10 to 50 cm above the ground surface, forwards and backwards (dashed black line). There are visible spots where the magnitude drops significantly. This is due to quick movement change of the UAV (e.g., usually when the flight direction has changed from forwards to backwards, or the opposite) and the antenna angle to the ground has changed. This means that during the scanning procedure it has to be ensured that the antenna to the ground surface angle remains the same. Figure 14b shows a closer look at a part of the obtained radargram. Shown is the real part instead of the magnitude, for the reason that a higher contrast was achieved. On the radargram, both landmines are clearly visible (dashed white line) as also are the antenna coupling (dotted orange line) and ground surface (dotted black line). Since a static object can cause strong backscatter and lower the visibility of other objects, averaging background subtraction was performed, and the result is shown in Figure 14c. We can see that parts such as the antenna coupling has been removed, and that ground backscatter is reduced in some regions. From the results, it is also visible that, in the scenario of a present landmine, not only the landmine itself is visible, but also the ground surface backscatter is modified, which gives additional useful information.

### 5.3. Performance Comparison between Other Systems

The developed system was compared to state-of-the-art GPR systems used on a UAV. Since the systems use different methods and setups, only the following main parameters which are relevant to us have been compared: Frequency range, power consumption and a total payload of the whole GPR system.

Table 2 shows selected parameters for comparison. Some of the reported parameters could not be determined, since they are not provided. These values have, therefore, been left empty, and they are excluded from the comparison. In order to give a better insight about the environment where the tests have been performed, a polygon description and experimental setup are summarized for each system:

- The SFCW GPR system: Inhomogeneous soil that contains smaller stones and is covered by grass, where test landmines have been buried down to 20 cm.
- System 1: tests have included AT and AP landmines, which are located above the ground surface on an outdoor polygon.
- System 2: tests have included corner reflectors with different Radar Cross Sections (RCS), metal cans and plastic boxes, which are located above the ground surface on an outdoor polygon.
- System 3: on-ground tests have been performed in sandy soil, where a metallic disk was buried down to 15 cm. In-flight tests have included a sandbox, where a metallic disk was buried down to 12 cm. The sandbox was covered with a canvas.

The parameter which influences the resolution of a radargram directly is the selected frequency range, which, furthermore, also defines the Bandwidth. Table 2 shows that System 2 uses the highest Bandwidth with B=3 GHz, followed by System 1, the SFCW GPR, and System 3. Here, the central frequency of the radar was not considered. Namely, this defines with which success the signal will couple with the propagation medium. Furthermore, output power and sensitivity also influence the radargram, but they could not be determined or they can not be directly compared between systems. Therefore, proper and comparable results could only be obtained with an evaluation of the GPR systems on the same polygon and under the same soil conditions.

By comparing the power consumption and total payload weight we can state that the SFCW GPR system shows better performance in both aspects. System 2 has not provided the power consumption, but even with comparable value to our proposed system, still the payload is more than twice the value. The radar module [29] of System 3 has lower power consumption, but as in the paper is stated an on-board computer (Raspberry Pi) is used to store acquired data. This furthermore increases the power consumption. System 1 has only provided the current consumption without mentioning the supply voltage.

## 6. Conclusions

The summary of results shows that the developed low power and weight SFCW GPR system is capable of detecting AP and AT landmines above or below ground using a basic processing strategy. Scanning at a height of about 0.5 m has still shown positive results, and, therefore, it makes the system usable for harsh environments where de-mining with ground-coupled systems would be nearly impossible. Experimental results do not define the maximum depth of a landmine or the maximum flight height clearly, when the system is still able to detect a landmine. This is not only due to the fact that, already, these two parameters are mutually dependent, but additionally, soil structure, moisture content, as also the landmine‘s size and material influence these values. Since we are in contact with demining experts of The Croatian Mine Action Centre (HCR-CTRO), by following their suggestions, the landmine detection depth was limited down to 20 cm, where the triggering probability is still high. The flight height of 0.5 m was selected with the purpose to avoid smaller objects or vegetation safely. AT landmines could also be detected from greater heights, but since the wish is to detect all of them with the highest possibility, both the depth and flight height have been limited to these selected values. Since soil moisture affects the detection, the aim is to perform scanning when the amount is at the lowest point possible. Therefore, dry and warm seasons are most suitable.

With power consumption of 4.2 W and board size 100 mm × 50 mm it is especially useful for UAV use or other systems where size, consumption or weight are critical limitations. Moreover, the ability to store data onboard internally has an advantage where no additional hardware is needed. By enabling the transmitter PA, scanning could be performed from higher distances by increasing power consumption, but this was not the objective of this work. The Vivaldi–Horn antenna has shown significant results with a combination of the SFCW GPR radar. The weight could be reduced additionally by using thinner plates for the signal waveguide and by optimization of mechanical parts. However, the antenna still represents the part that adds most of the weight and size to the system.

The scanning speed is now limited only by the frequency synthesizers, since they need a certain time to lock in the frequency. The speed itself could be increased by lowering the number of frequency steps, but this would increase the level of sidelobes in the radargram. The limitation could be solved in the hardware by adding multiple frequency synthesizers in parallel and switching between them.

## Figures and Tables

**Table 1:** Summary of SFCW GPR and Unmanned Aerial Vehicle (UAV) parameters.

Parameter	Value
Minimal freq. step	40 kHz
Maximal num. of freq. steps	53,750
Frequency range	550 MHz–2.7 GHz
TX output power	−4 dBm
Power Consumption	4.2 W
SFCW GPR size	100 mm × 50 mm
Antenna size (1 pcs)	95 mm × 225 mm × 180 mm
SFCW GPR weight	30 g
Antenna weight (1 pcs)	240 g
Total payload weight	780 g
UAV system autonomy	25 min

**Table 2:** Comparison of GPR system used for UAV purposes.

Parameter	SFCW GPR System	System 1 [12]	System 2 [13]	System 3 [14]
Frequency range	550 MHz–2.7 GHz	500 MHz–3 GHz	1 GHz–4 GHz	3.1 GHz–5.1 GHz
Power Consumption	4.2 W	3.5 A	/	2–3 W (only radar)
Total payload weight	0.78 kg	4 kg	1.6 kg	2–3 kg
